# Design and fabrication of a realistic anthropomorphic heterogeneous head phantom for MR purposes

**DOI:** 10.1371/journal.pone.0183168

**Published:** 2017-08-14

**Authors:** Sossena Wood, Narayanan Krishnamurthy, Tales Santini, Shailesh Raval, Nadim Farhat, John Andy Holmes, Tamer S. Ibrahim

**Affiliations:** 1 Department of Bioengineering, University of Pittsburgh, Pittsburgh, Pennsylvania, United States of America; 2 Swanson Center for Product Innovation, University of Pittsburgh, Pittsburgh, Pennsylvania, United States of America; 3 Department of Radiology, University of Pittsburgh, Pittsburgh, Pennsylvania, United States of America; Linköping University, SWEDEN

## Abstract

**Objective:**

The purpose of this study is to design an anthropomorphic heterogeneous head phantom that can be used for MRI and other electromagnetic applications.

**Materials and methods:**

An eight compartment, physical anthropomorphic head phantom was developed from a 3T MRI dataset of a healthy male. The designed phantom was successfully built and preliminarily evaluated through an application that involves electromagnetic-tissue interactions: MRI (due to it being an available resource). The developed phantom was filled with media possessing electromagnetic constitutive parameters that correspond to biological tissues at ~297 MHz. A preliminary comparison between an in-vivo human volunteer (based on whom the anthropomorphic head phantom was created) and various phantoms types, one being the anthropomorphic heterogeneous head phantom, were performed using a 7 Tesla human MRI scanner.

**Results:**

Echo planar imaging was performed and minimal ghosting and fluctuations were observed using the proposed anthropomorphic phantom. The magnetic field distributions (during MRI experiments at 7 Tesla) and the scattering parameter (measured using a network analyzer) were most comparable between the anthropomorphic heterogeneous head phantom and an in-vivo human volunteer.

**Conclusion:**

The developed anthropomorphic heterogeneous head phantom can be used as a resource to various researchers in applications that involve electromagnetic-biological tissue interactions such as MRI.

## Introduction

Phantoms are numerical and/or physical models that represent the characteristics of some specified human anatomy [1–4]. Phantoms are an inexpensive approach to testing several electromagnetic applications, specifically various medical diagnostic imaging tools and wireless communication applications [3, 5, 6]. Recent studies demonstrate how researchers use anthropomorphic phantoms in numerical and experimental studies as one of the many resources that help investigate the behavior of the interactions of electromagnetic (EM) fields and biological tissue(s) at varying electromagnetic frequencies [7]. While electromagnetic numerical modeling has been the greatest resource to understand and analyze the interaction of electromagnetic fields and biological tissue(s) [8–13], in the last few years, experimental phantoms are increasingly becoming a useful resource in conjunction with EM modeling [5, 14].

The design of physical phantoms has evolved over the years to verify the mimicry of a real patient or customer environment with the electromagnetic device in order to minimize the error in modeling the physical experiment. While the evolution and usage of physical phantoms is endless in electromagnetic applications, in this paper we will narrow our focus on how to develop and test a physical and realistic head phantom using magnetic resonance imaging (MRI/MR). We recommend a method to design and fabricate a physical anthropomorphic heterogeneous head phantom using 3D printing technology.

### Background

#### MRI applications

MRI phantoms are used to analyze, evaluate, and calibrate the MRI system and its instrumentation prior to conducting tests on humans. MRI phantoms also allow researchers to understand the phenomena of the interaction of electromagnetic waves and biological tissues most especially at high field strengths where these interactions are difficult to measure and to interpret [8, 15].

#### Prior work in developing electromagnetically-equivalent head-phantoms

Most designed and commercial phantoms are typically homogenous, simple in shape, and containing homogenous liquid. Today, most commercial whole-body MRI scanners provide a one-compartment spherical phantom filled with saline water. While studies supported by MR companies find homogeneous commercial phantoms to offer an acceptable quality assurance (QA) to test the MRI system, anthropomorphic shaped phantoms are typically needed to go beyond QA such as mimicking a human experiment. Furthermore, while commercially available homogenous phantoms are suitable resources for analysis and evaluation of lower field MRI systems, they are not typically viable for characterizing the electromagnetic-biological interactions at higher field strengths. At higher field strengths, the electromagnetic fields produced by MRI radiofrequency (RF) antennas become much more dependent on their interactions with biological tissues due to the higher operational frequency and consequently the RF wavelength is shortened [16].

In the early 2000s, studies [17–19] designed and used electromagnetic anthropomorphic homogenous head phantoms. An anthropomorphic homogenous head phantom, Specific Anthropomorphic Mannequin (SAM) [19], is a commonly used head phantom in many wireless communication application studies. The CHEMA [17] phantom and SAM were the most common physical anthropomorphic homogeneous phantoms used to quantify a real-life comparison of RF absorption. Safety protocols and standards of various electromagnetic committees within professional societies have approved physical homogeneous phantoms. Nonetheless, there is a need to research the feasibility of designing more anthropomorphic heterogeneous phantoms for applications that require accurate specific absorption rate (SAR) testing, analysis of the interaction of RF fields and biological tissue(s), and direct comparisons with in-vivo studies. IEEE Standards Association’s (IEEE-SA) standards and recommendations of IEEE SA—1528–2013 [19] recommends the criteria for the design of an anthropomorphic head phantom. Although study [19] states that heterogeneous head models are difficult to construct, studies [6, 14, 20] indicate the feasibility of constructing an anthropomorphic heterogeneous head phantom and build on the development of previous realistic heterogeneous phantoms [21–25]. The results of studies [6, 14, 20] support the finding [19] that heterogeneous phantoms are more accurate. Thus, various electromagnetic applications/ safety protocols/ standards will benefit from 1) describing the methodology of developing and 2) validating the results associated with, anthropomorphic heterogeneous head phantoms that can be tailored to a specific research lab and/ or an industry application.

Although to our knowledge the findings from Graedel et al [14] is the most comparative to this proposed work, the comparison of an anthropomorphic heterogeneous phantom to human head on which the phantom was prototyped does not exist. To further research the comparison mentioned above, this paper shares the findings of such a comparison with an increased number of compartments filled with biologically equivalent electromagnetic liquids. While the findings of studies [6, 14, 17] are helpful, there is a critical need to further develop anthropomorphic heterogeneous head phantoms so that researchers make realistic findings in various electromagnetic medical applications at varying frequencies.

#### Physical phantom construction: 3D printing

3D printing is becoming an attractive tool within the fields of medicine, science and engineering in a variety of applications. Specifically, within the bioengineering field, 3D bioprinting is commonly used in tissue and organ engineering [25]; and in the construction of bioprinted organs and anthropomorphic phantoms [26] of the human anatomy. Various imaging modalities (X-Ray, CT, MRI, etc.) can be incorporated to examine human anatomy and help in producing a physical model of the imaged tissue. Computer-aided design (CAD) software can also be used to accurately reproduce the imaged tissues from a file that contains the surface meshes and contours of the imaged tissues.

## Materials and methods

**Fig 1:**
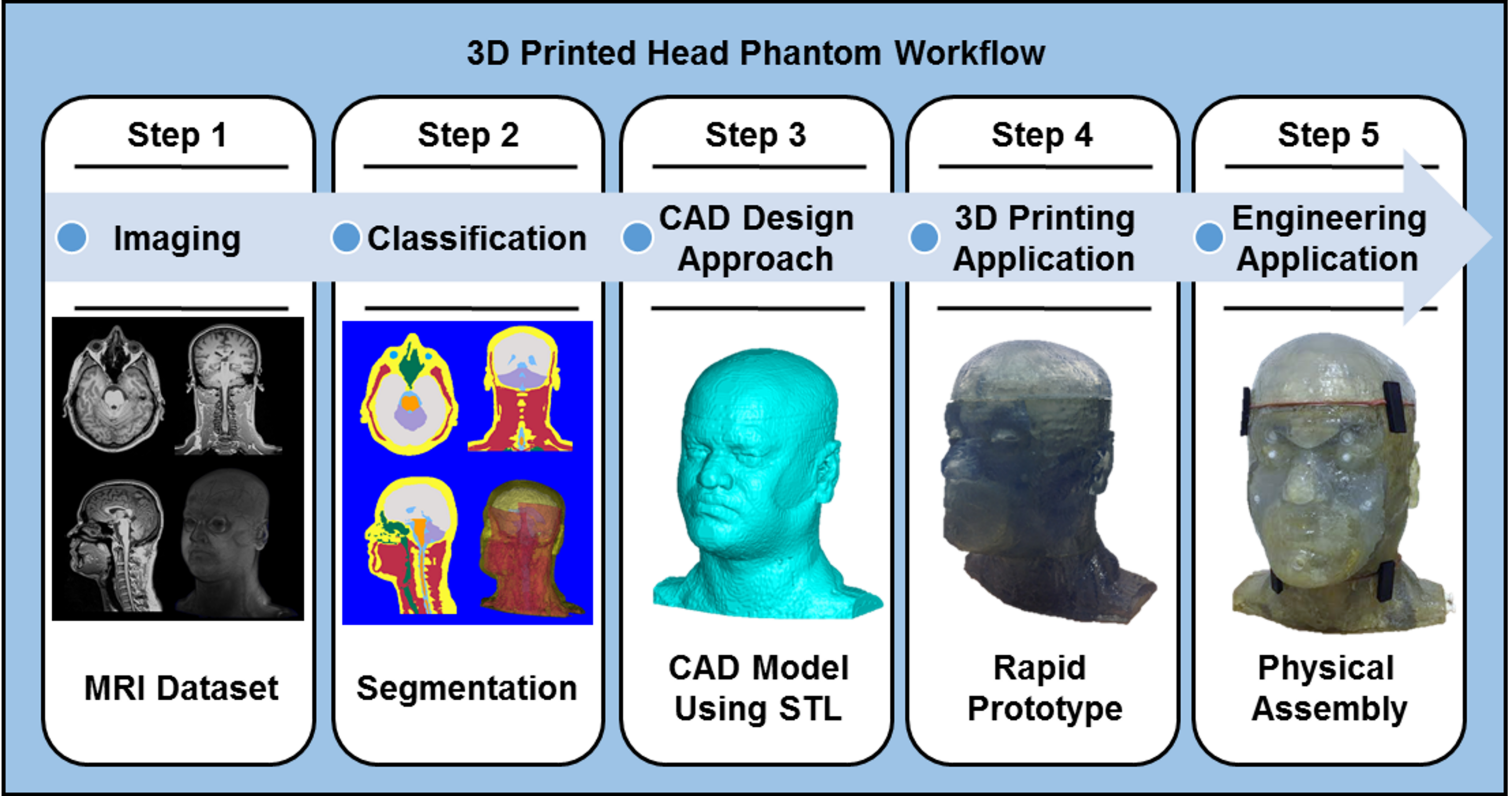
A General workflow to design and fabricate an anthropomorphic heterogeneous head phantom using 3D printing.

The design of the anthropomorphic heterogeneous phantom follows a workflow shown in Fig 1. The workflow is intended to be used for designing an anthropomorphic heterogeneous phantom of any physiological representation. This section outlines each step within the general workflow.

*Step 1*. *Acquiring 3D images of the human head*. The physical phantom’s application and functionality determines the choice of using a single or a combination of imaging modalities (i.e. MRI, CT, X-Ray, etc.)
*Step 2*. *Segmentation*. Segmentation–a design approach used to classify tissues within the imaged dataset–requires the developer to have the guidance of an expert in physiology or a physiological atlas in order to properly classify the tissues. Software that offers automatic segmentation algorithms should be used through most of the segmentation process. To obtain a geometric mesh of the classified tissues, the segmented data should be in a proper format that is compatible with CAD software and 3D printing software.
*Step 3*. *Modifying the design of the phantom for suitable 3D printing*. Specifications of 3D printing such as type and resolution should be considered during the design process to achieve a rapid prototype with limited errors.
*Step 4*. *Analyzing 3D printing techniques*. Issues regarding the 3D printing technology that is most suitable for the phantom should be considered. These include cost, material durability, compatibility of the material with the phantom’s application, and material printing resolution. Table 1 within study [27] provides a helpful summary of various 3D printers and each 3D printing materials’ corresponding characteristics.
*Step 5*. *Physical assembly of the head model*. Adequate steps for facilitating the design and assembly in the CAD software can minimize the extensive manual assembly efforts of the physical phantom.

**Table 1:** Constitutive properties and densities of the classified tissues at ~ 297 MHz.

Tissues		Electromagnetic Properties at Approximately 297 MHz		
Phantom Tissue Classification	General Biological Tissue Classification	Conductivity [S/m]	Relative Permittivity	Density [kg/m^3^]
Air	Sinuses, Esophagus	0.00	1.00	0.00
Brain (WM/GM)	White Matter, Gray Matter, External CSF, Dura	0.55	51.98	1040.00
Brainstem	Pons, Medulla Oblongata, Spinal Cord	0.42	36.97	1039.00
Cerebellum	Cerebellum	0.97	59.86	1040.00
Cerebrospinal Fluid (CSF)	Internal CSF near lateral horns inside the ventricles and around spinal cord	2.22	72.80	1007.00
Eyes	Cornea, Vitreous Humor, Eyes Sclera	0.92	56.46	1020.07
Muscle	Tendons, Tongue, Muscle	0.77	58.24	1049.78
Fat/ Bone/ Skin (3D SLA Material)	Fat, Bone, Cartilage, Skin	0.11	6.18	1120.00

The following represents the developmental steps for developing our anthropomorphic heterogeneous human head phantom.

### 1. Image acquisition

An MR dataset is acquired using 3D magnetization-prepared rapid gradient-echo (MPRAGE) sequence because of its ability to offer excellent structural contrast in order to appropriately segment various tissue [23, 28, 29]. The MR images were acquired from a healthy adult, male, human volunteer using a Siemens MAGNETOM TIM Trio 3T whole-body scanner (Erlangen, Germany) at University of Pittsburgh Medical Center. The images are isotropic, T1-weighted with the following parameters: FOV: 320x320 mm^2^; TE: 2.62 ms; TR: 2110 ms; TI: 1100 ms; FA: 8°; BW: 200 Hz/pixel; Resolution: 1.0x1.0x1.0 mm^3^).

### 2. Phantom segmentation, design and fabrication

**Fig 2:**
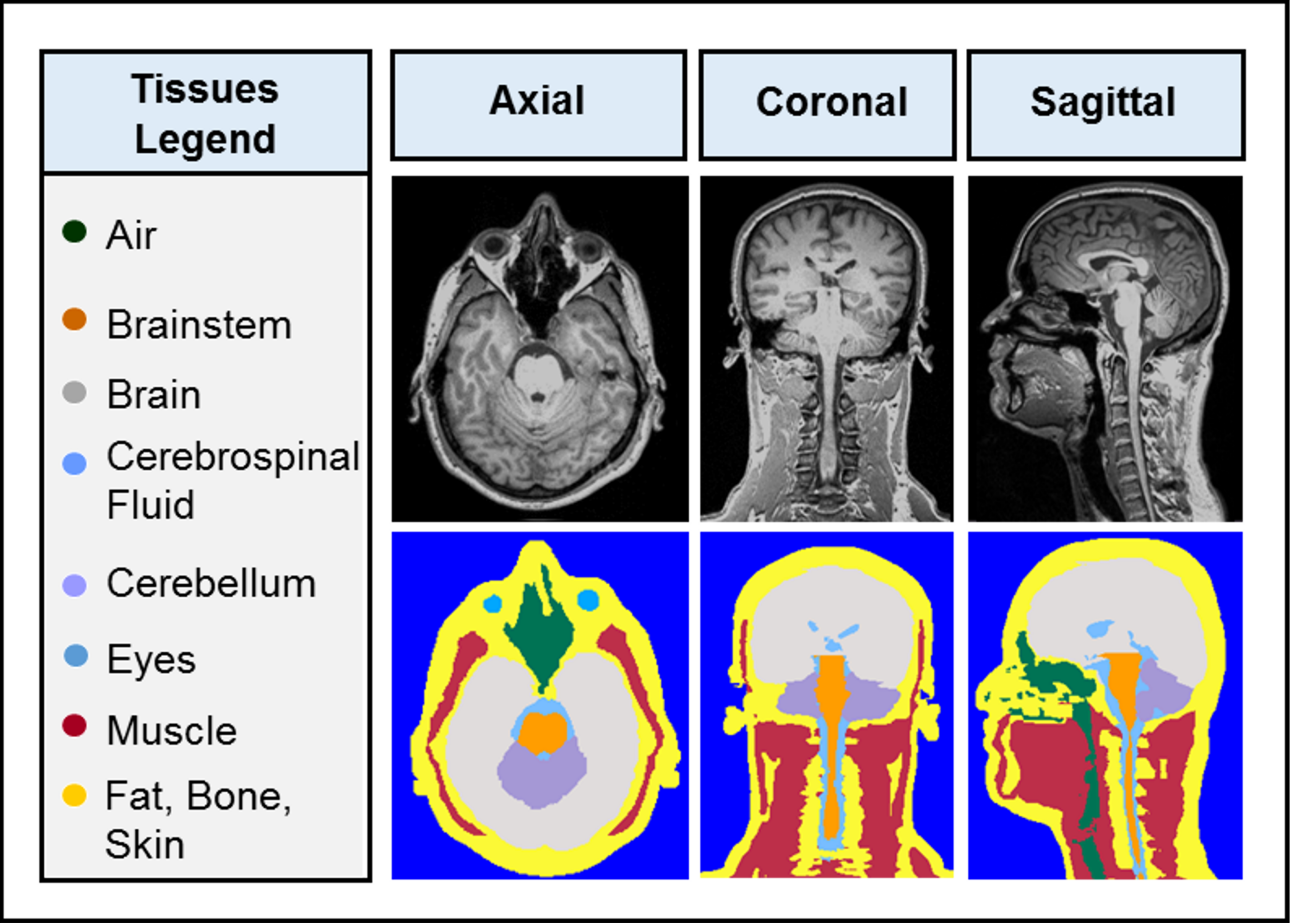
Medical data acquisition and segmentation. A 3T MRI scan with 1.0x1.0x1.0mm^3^ resolution was segmented and divided into eight individual tissues. Each segmented tissue is listed with the corresponding tissue segmentation color within the tissue legend. The pictured MRI dataset and segmented tissues are shown in the mid axial, coronal and sagittal views. Table 1 lists the physiological tissues that were used to classify the tissues in the legend.

An eight-tissue compartment head phantom was segmented and developed from the 3T MRI dataset as shown in Fig 2. To obtain an anatomically detailed human head numerical model, tissues were labeled and automatically segmented using iSeg (ZMT Zurich MedTech AG, Zurich, Switzerland) segmentation software. To achieve accurate labeling, a human head atlas [28] was used to properly classify the tissues and segment the dataset as shown in Fig 2. The head phantom compartments consist of eight grouped classified tissues namely: brain, brainstem, eyes, air cavities, cerebellum, cerebrospinal fluid (CSF), muscle, and the remainder volume being a combination of the fat, bone, and skin. The classification of the tissues was distinguished by relatively similar constitutive parameters and the ability to fabricate the model. We combined, for instance, the white matter, grey matter and the CSF—in the vicinity of the grey and white matter—and classified the grouped tissue as “brain”. Similarly, we used the same logic for the “eye”, which is a combination of the physiological tissues known as the lens, vitreous humor, cornea and sclera. Once classified, the tissues were exported from iSeg as surface mesh objects in STereoLithography (STL) format and voxel matrices in MATLAB (mat) format.

**Fig 3:**
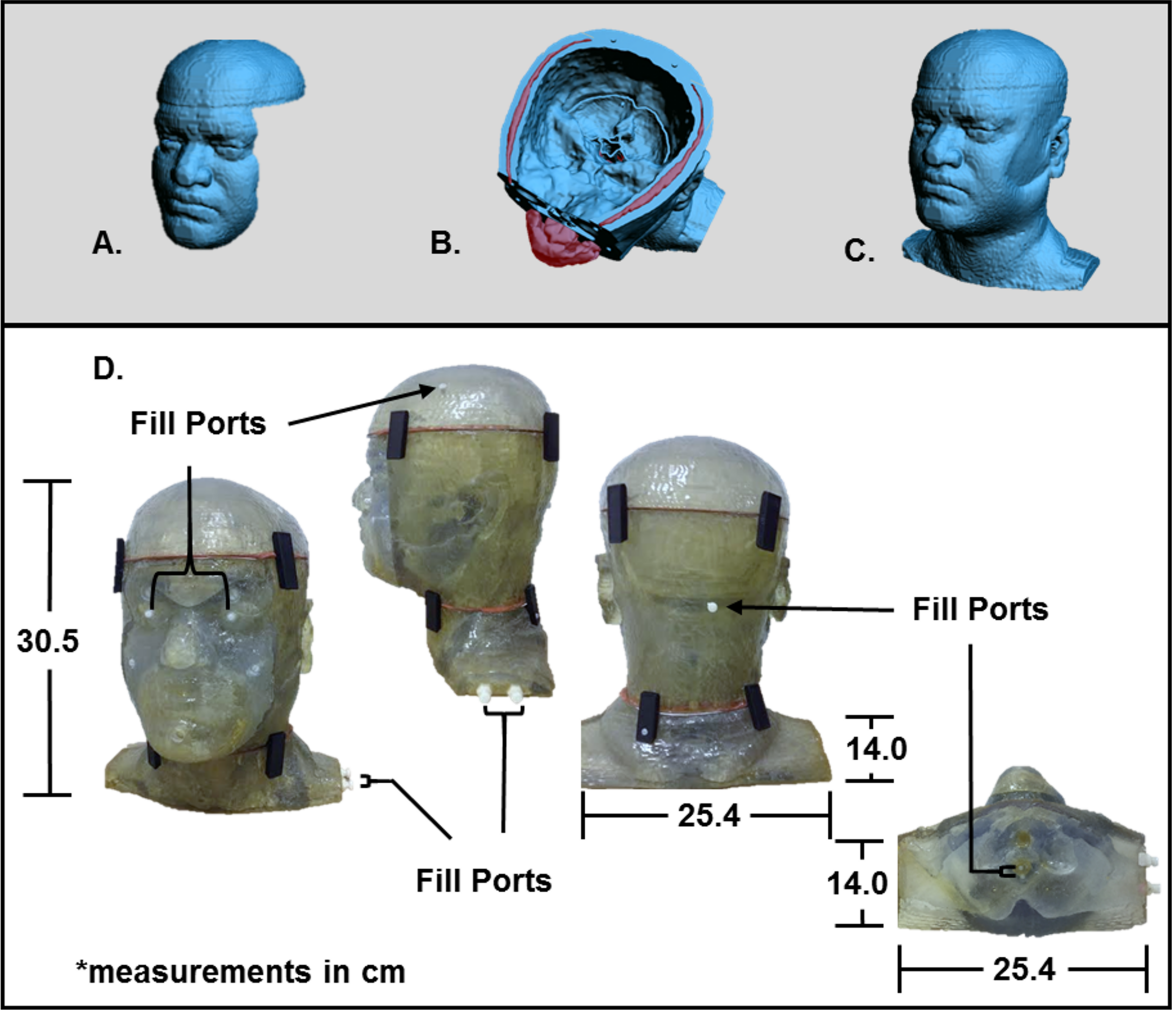
Design and fabrication of physical phantom model. Views of the shelled CAD files (A-C) which were developed in order to make volumetric cavities of the designated biological tissues that were segmented from a 3T MRI dataset. Views of the rapid prototype model (D) show the head phantom printed with stereolithography (SLA) resin. The physical head phantom dimensions are 30.5 cm tall, 25.4 cm long and 14.0 cm wide. The filling-ports are highlighted by arrows indicating the locations at which the fluids, resembling various tissue types, enter the phantom.

Using 3D CAD software (Geomagic Studios 2012 (Geomagic, Morrisville, North Carolina)), each compartment was designed to reserve the mixture of the desired tissue over time as seen in Fig 3A–3C. The wall thickness of each tissue compartment and the molding compartment—combination of fat, bone and skin—were rendered and later printed using stereolithography (SLA) resin (DSM Somos WaterShed XC 11122 (Elgin, Illinois)) with the Stratasys 3D printer (Stratasys, Eden Prairie, Minnesota). Six compartments are refillable with fluid through chambers that were positioned within each of the designated tissue compartments. The other two compartments are the molding compartment and the air cavities.

The cured SLA resin material is hydrophobic and durable to external and internal pressures. Among all of the 3D printing materials available, the DSM Somos WaterShed XC 11122 best fits the need to preserve the liquid with time. We tried polycarbonate material in the fabrication of a single-compartment, anthropomorphic homogenous head phantom. The polycarbonate material is not hydrophobic and it is porous. With time, the liquid evaporated from the inside and crystalized on the exterior of the model. This material, however, was useful for the study [20] that utilized ABS material and a waterproof spray coating or other studies that used agar to mimic the biological tissues. In contrast, an application of waterproof spray coating is unnecessary with the SLA material used in this study.

The physical head phantom model was designed and printed in five separate parts in order to manually remove the inner structural supports that come out with 3D printing. Leaving the structural supports inside of the model is undesired because it causes artifacts and bubbles within each tissue compartment leading to less accurate approximation of the electromagnetic fields produced in biological tissues.

### 3. Physical assembly

Once the model was 3D printed, manual mechanics were applied to each printed part. After the structural inserts were removed, each part was manually smoothed by sanding in areas where the printed parts do not mesh well. Using a combination of sandpaper and the Dremel tool (Robert Bosch Tool Corporation, Mt. Prospect, Illinois) is typically necessary. The overall print time of the phantom took almost a week in hours.

To make the physical model fully airtight, various engineering designs were considered and the final phantom design incorporated epoxy adhesive, plastic latches, nylon screws and threads, and rubber foam. A well-designed CAD model can reduce manual construction. Various sealants were researched for their electromagnetic compatibility as well as MR signal. Materials with metallic components were avoided. An epoxy adhesive (DP100 Plus Clear, 3M Scotch-Weld, St. Paul, Minnesota) was applied to various halves to bind the physical 3D printed model parts together. Epoxy adhesive was only used on parts that would not be reopened. For parts that required reentry, the silicone rubber was applied as a sealant and then doubled on contacting surfaces to act as a rubber gasket. Latches were strategically mounted on the physical phantom model to apply pressure to the foam as sealant, which prevents the filling liquids from leaking at these junctions.

The filling ports were manually designed based on the CAD model and the ease of filling the designated liquid per compartment. Each refillable liquid has at least two ports: one for filling, while the other for releasing air bubbles that may accumulate during the filling process.

### 4. Preparation of the phantom filling

In this study, the tissues in Table 1 were classified as biological tissues that are grouped together based on the location and constitutive parameters of the tissue. These groups were chosen to represent tissues of the phantom without running into the complications of having an overly detailed and ineffective design with diminished structural integrity. While the physical model is realistic, some assumptions were made to match the constitutive properties of the human tissues listed by Gabriel et al. [29]. These values are based on calculations of the constitutive parameters while being a function of the physiological tissues density that make up each group. The air cavities are representative of the nasal cavities, sinuses and esophagus. The brain tissue composes of the gray matter, white matter, blood vessels in the brain, Dura and the CSF in between and surrounding the brain. The brainstem is representative of the pons, medulla oblongata and the spinal cord. The constitutive parameters of the brainstem tissue match those of the spinal cord listed in [29]. The internal CSF is representative of the actual CSF inside of the ventricles and lateral horns—lateral gray columns of the spinal cord—and the CSF that surrounds the exterior of the spinal cord. The cerebellum is representative of the actual cerebellum. The eye tissue is a composition of the vitreous humor, cornea, eye sclera, nerves and the blood vessels within the eye. The muscle is representative of the continuity of muscle and tendons in the head (including the tongue), neck and upper shoulders. The bone is representative of the bone, cartilage, bone marrow and bone cortical throughout the head, neck and upper shoulders. Since the bone possesses relatively lower values in terms of conductivity and permittivity and the fat is a discontinuous tissue with similar electromagnetic characteristics, the phantom combines both the bone, fat, and skin (not considered in this study) into one tissue. This combined tissue is physically and electromagnetically representative of the SLA resin material due to each corresponding low loss/permittivity (with the exception of the skin). The skin was too thin to be properly segmented and 3D printed into a separate tissue for filling.

Table 1 provide values that were achieved by calculations using studies [23, 29]. The conductivity (σ) and permittivity (ϵ_r_) volumes were developed by in-house mixtures of distilled water, sodium chloride (NaCl), and/or denatured ethanol (C_2_H_6_O) at room temperature. The in-house mixture must have a relatively low viscosity while remaining soluble; thus, the selected chemicals are used for a relatively inexpensive in-house mixture. The constitutive parameters of each tissue shown in Table 1 [29] were measured using a dielectric probe (SPEAG DAK (AG SPE, Zurich, Switzerland)) with measurements calibrated between a spectrum window of 295 MHz and 300 MHz. Distilled water was chosen as a base for the solution within the six compartments. Various studies [29, 30] demonstrate that the permittivity value of water decreases as concentrations of solvents with lower permittivity values are mixed into the solution. NaCl was used to control the conductivity [31] and C_2_H_6_O was used to adjust the permittivity of the developed solution. Various concentrations of NaCl and C_2_H_6_O were used to match the values that are listed in [29]. Using the dielectric probe, the values reported in Table 1 were measured several times (n = 10, σ = 0.01) to ensure stability over time. The prepared liquids with constitutive parameters shown in Table 1 were used to fill the phantom.

### Network analyzer measurements

Workbench analysis of the three phantoms and an in-vivo volunteer centered in an RF coil were measured with a network analyzer (Agilent E5602A, Keysight Technologies, Santa Rosa, California). Scattering parameters indicate to an RF engineer how well tuned/matched a coil is to the present load with measurements of transmission and reflection per channel. Using the scattering parameters, each phantom and the volunteer were characterized through the reflection coefficient (S11) of one representative channel and therefore the input impedance of an in-house built RF volume coil (a 16-strut transverse electromagnetic (TEM) resonator) [32, 33].

### 7T MRI experiments

Experimental B_1_ mapping of the anthropomorphic heterogeneous head phantom, anthropomorphic homogeneous head phantom, spherical phantom and the in-vivo volunteer (with signed consent form approved by the Institutional Review Board at the University of Pittsburgh) within the TEM coil were acquired using Siemens MAGNETOM 7T whole-body scanner (Erlangen, Germany). The volunteer has given a written informed consent (as outlined by PLOS ONE consent form) to publish the details in this manuscript. The anthropomorphic homogeneous head phantom (the designed phantom) and the spherical phantom were filled with a solution that has conductivity = 0.41 S/m and relative dielectric constant = 79. The in-vivo study was performed by acquiring images with the head centered within the TEM coil. The sequence used for B_1_ mapping was SAT TurboFLASH with the following parameters: Pulse: rectangular RF pulse of 1 ms at 500V; FOV: 64 x 64 mm^2^; TE: 1.16 ms; TR: 2000 ms; FA: 6°; BW: 1502 Hz/pixel; and Resolution: 3.1 x 3.1 x 2.0 mm^3^.

The stability of the head phantom and in-vivo volunteer were measured using similar stability QA protocols methods used at lower field strengths [34]. The RF shielding in this particular TEM resonator produces ghosting effects and is very distorted; thus, another commercially available RF head coil was used to properly evaluate echo planar imaging (EPI) stability scans. Experimental 2D EPI images of the anthropomorphic heterogeneous phantom and the in-vivo volunteer were acquired by centering the head phantom and the volunteer within the 8-channel Rapid coil (Rapid Biomedical, Wurzburg, Germany). The sequence for EPI acquisition used the following parameters: FOV: 148 x 148 mm^2^; TE: 20 ms; TR: 2500 ms; FA: 65°; BW: 1778 Hz/pixel; Acquisitions: 10; Slices: 86; Scan Plane: Axial; and Resolution: 1.5 x 1.5 x 1.5 mm^3^. QA data was analyzed quantitatively through fBIRN (NA-MIC, Bethesda, MD, USA) and Matlab (The MathWorks Inc., Natwick, MA, USA). In this work, we monitored the stability through the signal-to-ghosting ratio (SGR) and fluctuation indicated by Friedman and Glover [35, 36] in one comparative slice for 9 acquisitions (similar to typical fMRI analysis, the first acquisition was removed from the analysis). The SGR is computed using eq. (2) in Simmons [37] and Weisskoff [34] and the fluctuation using eq. (5) in Simmons [37] is applied.

## Results

### Fabrication of the head phantom

The phantom was successfully fabricated as shown in Fig 3D which demonstrates the feasibility of using 3D printing technology to develop an anthropomorphic heterogeneous head phantom. The in-house mixtures are made with low viscosity and it requires no more than an hour to fill the entire phantom with limited air bubbles. Emptying the phantom is much quicker; however, there are multiple methods to empty the phantom. Both ports must be open and the liquid can be poured out or suctioned out based on preference.

### S-Matrix measurements of the phantoms and the in-vivo volunteer

**Fig 4:**
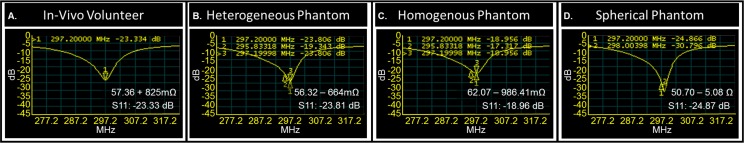
Comparison of phantoms to in-vivo volunteer using the scattering parameters of an RF coil.

The scattering parameters of each phantom were successfully measured and shown in Fig 4. The reflection coefficients for each phantom (Fig 4A–4C) and in-vivo volunteer (Fig 4D) at 297.2MHz are listed respectively: -23.33 dB, -23.81 dB, -18.96 dB, and -24.87 dB. The resonant frequency of 297.2 MHz is indicated by marker 1.

Fig 4B–4D represent the shift in resonance of the heterogeneous phantom (Fig 4B), homogeneous phantom (Fig 4C) and spherical phantom (Fig 4D), in comparison to the in-vivo volunteer (Fig 4A) through markers 2 and 3. The coil is tuned to the volunteer at 297.2MHz (indicated by marker 1) and has an input impedance of 57.36 + 825mΩ. The heterogeneous head phantom shifts (0.1MHz) to the right with an input impedance of 56.32–664mΩ. The homogeneous head phantom shifts (0.1MHz) to the left with an input impedance of 62.07–986.41Ω and the spherical phantom (0.8MHz) shifts to the right with an input impedance of 50.70–5.08Ω. The bench measurements indicate that the heterogeneous head phantom is most comparable to the in-vivo volunteer in terms of input impedance.

### Experimental measurements of various phantoms and the in-vivo volunteer

**Fig 5:**
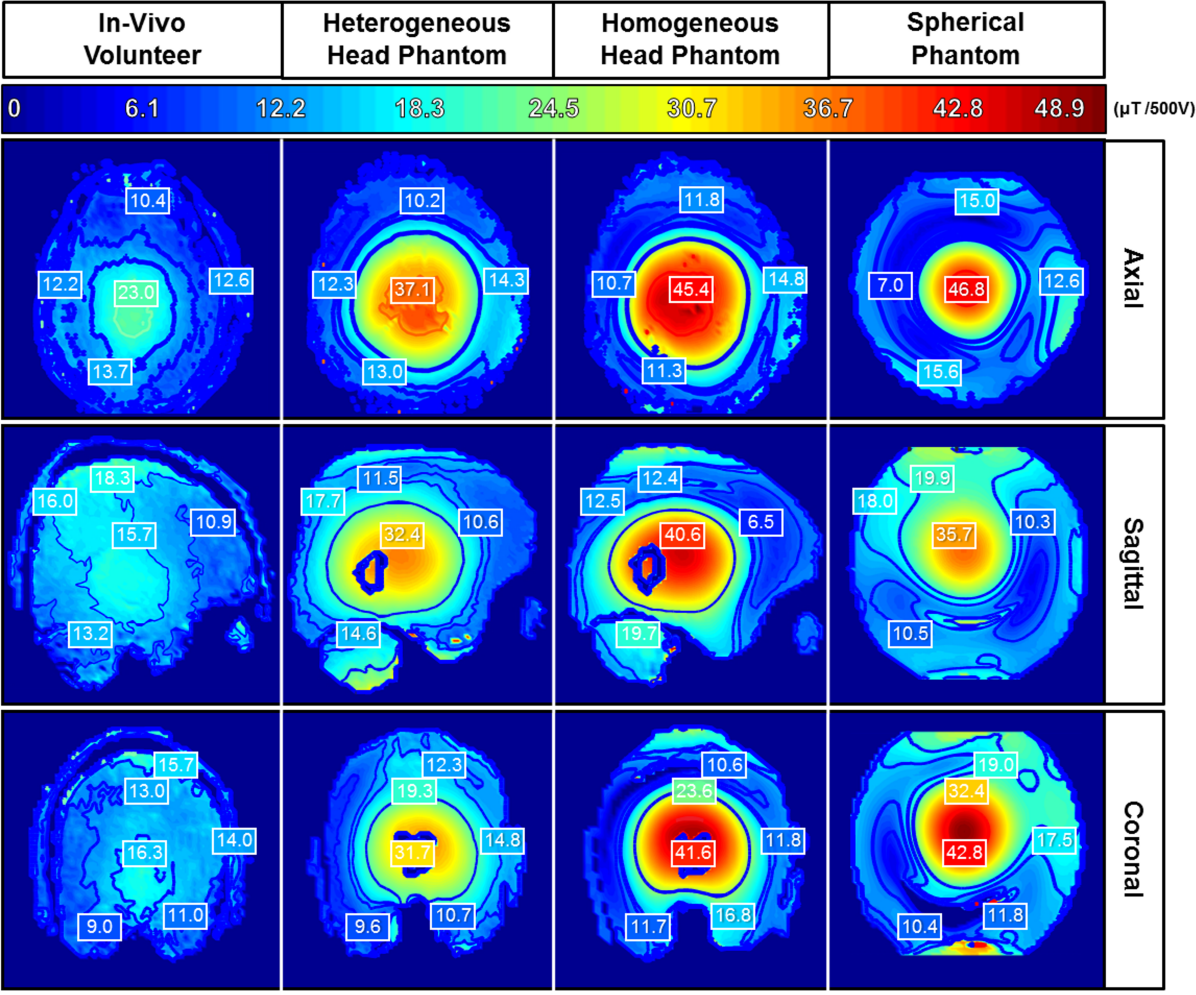
Comparison of the experimentally mapped magnetic (B_1_) field distributions. Congruent slices of each phantom in comparison with the human volunteer are shown in all planar views. The color bar ranges from 0 to 48.9μT per 500V. The maximum B1 intensity level is set to the highest pixel value among each of the phantoms and the volunteer.

The experimental B_1_ mapping statistics and distributions of both head phantoms, spherical phantom and the in-vivo volunteer are captured for one axial, coronal and sagittal slices at comparable locations as shown in Fig 5.

The results shown in Fig 5 represent a preliminary evaluation of the electromagnetic behavior of the three phantoms and the in-vivo volunteer. Each B1 map is computed by $B_{1}=\frac{\theta }{\mathrm{\gamma t}}$, where *θ* is the flip angle, *γ* is the gyromagnetic ratio = 42.58 MHz/T, B1 is the RF magnetic field (at 7T or 297 MHz) and t is the pulse width. The B_1_ was acquired and scaled to a voltage of 500V for a rectangular pulse duration of 1ms. Qualitative/quantitative analysis were conducted along congruent slices of each phantom in comparison with the human volunteer. The contours span the same range in terms of values.

When compared to the other two phantoms, the anthropomorphic heterogeneous head phantom possesses the most comparable B_1_ field distribution to the in-vivo volunteer. The other phantoms have a higher magnetic intensity per volt when compared to the anthropomorphic heterogeneous head phantom and the human volunteer.

**Fig 6:**
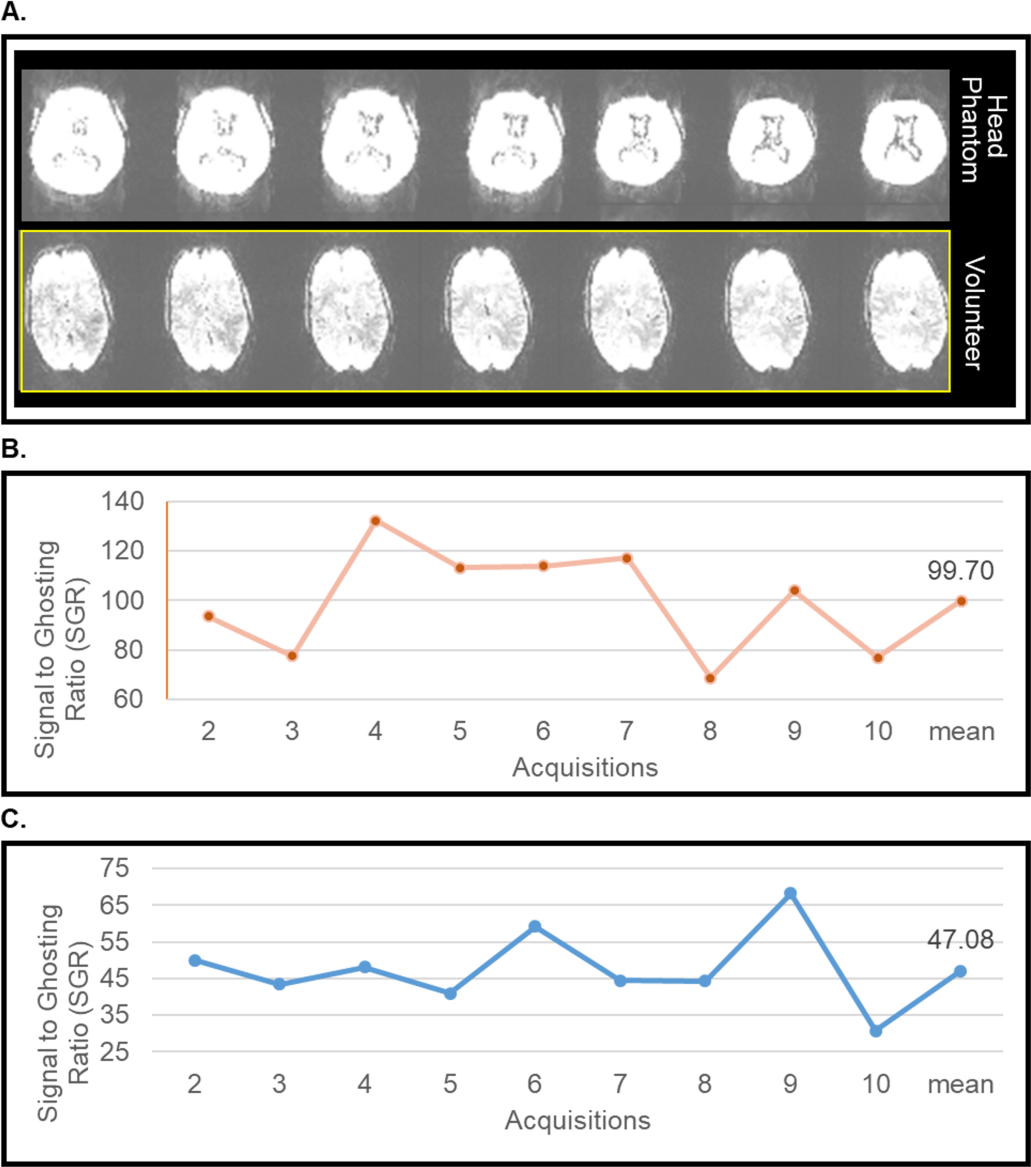
Comparison of phantom to in-vivo volunteer during an EPI stability scan at 7T MRI.

The results shown in Fig 6 represent an evaluation of EPI stability scans of the anthropomorphic heterogeneous phantom, and the volunteer. The stability parameters such as the SGR and fluctuation are shown in Table 2. The contrast in the images of the anthropomorphic heterogeneous phantom and the volunteer is adjusted to show the ghosting (Fig 6A). Since the phantom does not experience movement, comparing the stability of the phantom and the volunteer presents some difficulty; nonetheless Fig 6 presents SGR in the heterogeneous phantom (Fig 6B) and the volunteer (Fig 6C) over 9 EPI measurements.

**Table 2:** Stability parameters of phantoms and the volunteer for the EPI stability scans at 7T MRI.

Stability Parameter	Head Phantom	Volunteer
**SGR**	99.7	47.1
**Fluctuation (%)**	0.10	4.16

## Discussion

This work aims to address the challenges associated with phantom design and fabrication and offers a methodology to design, fabricate and evaluate the development of a realistic anthropomorphic heterogeneous head phantom for various electromagnetic applications. The design and construction of the head phantom differs from homogeneous [15, 17, 38] phantoms and recent anthropomorphic heterogeneous [6, 18, 21, 22, 24] phantoms as it is more comparable to a volunteer from whom it was developed. The design is one solution to the challenge of making a heterogeneous physical phantom.

### Fabrication of the head phantom

The phantom was determined to voxelized and print the phantom at a higher printing resolution. It was determined that smoothening the CAD models as an automated process was not desired since it would remove the detail of the phantom and lessen the accuracy of the electromagnetic comparison to the volunteer. Thus, the physical assembly of the phantom took a few months to complete and required extensive planning.

### S-Matrix of the various phantoms and the in-vivo volunteer

The bench analysis demonstrates that the anthropomorphic phantom is a useful tool for RF engineers in order to conduct loading analysis for RF coil developments. From the current measurements and in terms of input impedance, both anthropomorphic head phantoms appeared more realistic than the spherical phantom when compared to the volunteer.

### Magnetic field distributions and EPI testing of the various phantoms and the in-vivo volunteer

The B_1_ field distribution for each phantom and the volunteer does not have uniform distribution in any planar view. The B_1_ maps highlight the phenomena of a shorter RF wavelength at higher field strengths [39–42]. The B_1_ mapping results demonstrate that the anthropomorphic heterogeneous head phantom is the most realistic phantom to mimic and model the in-vivo volunteer. While our anthropomorphic heterogeneous head phantom does not offer full accuracy in its comparison of the electromagnetic fields to the in-vivo volunteer, it is considerably more comparable than the spherical, anthropomorphic homogenous head phantom or other fabricated heterogeneous phantom studies [6, 14]. The magnetic field distribution of the spherical homogenous phantom is most comparable to the anthropomorphic homogenous head phantom. The B_1_ intensity values are higher in the phantoms due to having differing constitutive parameters for the skin tissue resulting in higher RF penetration and therefore higher B_1_ values. That being said, the heterogeneous phantom is the most comparable to the in-vivo volunteer in terms B1 distribution and intensity. As noted, while the anthropomorphic homogeneous head phantom has the same molding and contours as the anthropomorphic heterogeneous head phantom, it uses the same constitutive parameters as the spherical phantom. More evaluations need to be conducted in order to determine how the phantom’s anatomy and the thickness of the compartment shells affect the electromagnetic field distributions.

Short acquisitions are usually carried out to demonstrate stability testing. We have compared the stability parameters in Table 2 of the anthropomorphic heterogeneous head phantom and the volunteer. As expected, the fluctuation indicates that the phantom’s signal is stable compared to the volunteer. The difference between the mean SGR of the head phantom and volunteer are about 112%. Because the phantom does not offer comparable bold contrast to the volunteer, the signal intensity is much higher in the phantom.

Although, we achieved success segmenting, designing and fabricating the phantom, the phantom design presents limitations to some electromagnetic applications. The representation of the fat, bone and skin as one tissue is a limitation. Due to this limitation, the particular tissue will have an artificially lower SAR. In various telecommunications applications, the SAR is observed to be the highest in the ear for adult models. The electromagnetic properties of the physiological skin and ear are more conductive than the SLA resin material. The exact comparison and measurement of this limitation can be evaluated in future investigations.

### Future phantom applications

Our study has the potential to span and benefit many EM applications. While numerical EM modeling is still a helpful resource for various EM applications, our study indicates that there is a benefit to further developing physical phantoms to study the interaction of EM waves and biological tissue in the real experimental environment.

There is an opportunity to evolve this study further to assess the benefit of using a realistic physical phantom in MR applications. There are a variety of MR applications that would benefit from using a physical phantom. Perhaps, the most beneficial applications are those that involve a true assessment of RF safety. Future studies using the designed phantom will include MR thermometry, RF coil design, and implanted devices.

As previously mentioned, SAM is a commonly used head phantom in many wireless communication application studies. Many studies [5, 43–45] use the SAM phantom along with a hand phantom to evaluate the emitted RF signals of a wireless RF antenna (i.e. a cell phone, Bluetooth device, Google glasses, etc.) and the SAR within the tissue in relation to government regulated standards for health concerns. From the results of our study and other studies [6, 14, 20], we recommend utilizing heterogeneous phantoms to conduct RF testing.

Although we designed the phantom for MR purposes, the design and fabrication of the phantom can be used for various EM applications.

## Conclusion

An anthropomorphic heterogeneous head phantom based on in-vivo MR dataset was developed, tested and compared to a human head.

## Supporting information

S1 Multimedia Visual representation to construct a physical head anthropomorphic heterogeneous phantom. A visual representation of the 3D printed workflow in Fig 1. This video highlights each step in the process to create an anthropomorphic phantom.
(MP4)
