# Correction: Design and fabrication of a realistic anthropomorphic heterogeneous head phantom for MR purposes

**DOI:** 10.1371/journal.pone.0192794

**Published:** 2018-02-07

**Authors:** Sossena Wood, Narayanan Krishnamurthy, Tales Santini, Shailesh B. Raval, Nadim Farhat, John Andy Holmes, Tamer S. Ibrahim

The fourth author’s name is spelled incorrectly. The correct name is: Shailesh B. Raval.

The correct citation is: Wood S, Krishnamurthy N, Santini T, Raval SB, Farhat N, Holmes JA, et al. (2017) Design and fabrication of a realistic anthropomorphic heterogeneous head phantom for MR purposes. PLoS ONE 12(8): e0183168. https://doi.org/10.1371/journal.pone.0183168.

An affiliation for the fourth author is missing. Shailesh B. Ravel is affiliated with both #1 and #3 Department of Radiology, University of Pittsburgh, Pittsburgh, Pennsylvania, United States of America.
